# Palatal dimensions at different stages of dentition in 5 to 18-year-old Iranian children and adolescent with normal occlusion

**DOI:** 10.1186/s12903-018-0538-y

**Published:** 2018-05-15

**Authors:** Gholamreza Eslami Amirabadi, Amin Golshah, Sepideh Derakhshan, Shahla Khandan, Mahshid Saeidipour, Nafiseh Nikkerdar

**Affiliations:** 1 Orthodontic Department, School of Dentistry, Shahed University, Tehran, Iran; 2 Orthodontic Department, School of Dentistry, Kermanshah University of Medical Sciences, Kermanshah, Iran; 3 Hamedan University of Medical Sciences, Hamedan, Iran; 4 Kermanshah University of Medical Sciences, Kermanshah, Iran; 5 Oral and Maxillofacial Radiology Department, School of Dentistry, Kermanshah University of Medical Sciences, Kermanshah, Iran

**Keywords:** Palate, Dimension, Dentition, Dental arch, Maxillofacial development

## Abstract

**Background:**

This study was purposed to evaluate palatal width, height, and height index at various stages of dentition in Iranian children and adolescent with normal occlusion.

**Methods:**

This cross-sectional study was conducted on a random sample of 237 children (45% male and 55% female, aged 5–18 years) with normal occlusion selected from kindergartens and elementary and high schools in Hamadan, Iran. The subjects were clinically examined and classified based on dentition to primary (21.5%), mixed (21.9%), and permanent (56.5%) stages. Dental casts were obtained from all subjects. Palatal width (inter-molar and -canine distances), and height (at molar and canine areas) were measured on the casts by Korkhaus’ compass and digital caliper. Palatal height index was calculated for each dentition stage. Data were analyzed by SPSS 15 using one-way ANOVA followed by Tukey’s post hoc test and t- test (*p* <  0.05).

**Results:**

Palatal inter-molar and -canine width values were increased from primary to permanent dentition. Palatal height and palatal height index in mixed dentition were significantly lower than those in primary and permanent dentition. Palatal width at inter-molar and -canine distances was significantly higher in males than females. There was no significant difference in palatal height and palatal height index at molar area between males and females. However, palatal height and palatal height index at canine area were significantly higher in males compared to females.

**Conclusion:**

These findings showed that palatal width increased from primary to permanent stage. Palatal height and palatal height index decrease from primary to mixed dentation, then increase from mixed to permanent dentition.

## Background

The human dentition [1] and dental arch dimensions [2] continuously change up to adulthood. Changes in facial dimensions occur in vertical and sagittal dimensions throughout the growth [3]. During transition of dentition, dental arch form and width alter due to tooth movement and vertical growth of alveolar process [4–7].The shape and size of the process are influenced by alterations in different parts of the skull [8]. The size and shape of dental arches vary in different stage of dentition that should be considered for orthodontic diagnosis, treatment and stability of treatment out-comes [1]. The changes that occur in the dental arches in these periods of mixed dentition are the result of tooth movement and growth of the supporting bone [9]. Changes of palatal width take place in both canine and molar sites from childhood to adolescence [10]. Numerous studies have reported an increase in palatal inter-canine [1, 7, 10–16] and inter-molar [1, 10–23] width from primary to early permanent dentition. Eruption of the permanent incisors resulted in an increase of the width and high of anterior segment of the maxilla. Alveolar processes diverge as the teeth erupt. A further minor increase of palatal width and height was occurred with eruption of the permanent canines [1–3, 10].

Palatal height changes as a child grows up. The palatal height increases continuously, with a higher rate between 5 to 16 years of age [1]. However, there are few studies investigating the palatal height in normal occlusion [1, 18, 24].

The palatal height index is obtained from the combination of the dimensions of height and width which was first introduced by Korkhaus [25]. There are few reports of palatal height index changes during growth in normal occlusion [18, 26, 27].

Sex may play an important role in determination of palatal dimensions and their changes during developmental growth. Amount of increase in palatal dimensions has been reported to be greater in males than in females [10, 11, 28].

The palatal changes have had considerable implications in orthodontic diagnosis and treatment planning in a modern dentistry based on prevention and early diagnosis of oral disease that orthodontists should accurately take into consideration. The naturally occurring changes of arch dimensions during growth have been used as comparative “gold standards” to distinguish changes induced by appliance therapy. These changes have been employed to assist the diagnosis, orthodontic planning and post-retention stability [2, 10, 29].

Moreover, knowledge about normal values of palatal dimensions can be used as a baseline for studies on oral developmental abnormalities [24].

It has been reported that palatal dimension is influenced by ethnicity [28], dietary regimens [30] and environmental factor [10]. Each population affinity and ethnic group possess its own specific facial and cranial form [31, 32]. For example, Iranians have wider nose, philtrum and lower face width(inter-gonial distance), narrower mouth, and shorter nose length than North American whites [31, 33, 34]. Iranians tend to have an increased ANB (A point-Nasion-B point) angle, IMPA (incisor to mandibular plane angle), bimaxillary protrusion, facial convexity, upper lip thickness, and soft tissue chin thickness as compared with published norms [35–37]. To the best of our knowledge, there is no study on palatal dimension norms and changes during growth in Iranian population with normal occlusion. Therefore, this study was aimed to evaluate the palatal width, palatal height and palatal height index at different stages of dentition in 5 to 18 year-old Iranian children and adolescent with normal occlusion.

## Methods

This cross-sectional study was carried on children with normal occlusion from Hamadan, Iran. Signed informed consent was obtained from each subject’s parents.

A sample of 150 (50 for each stage) was estimated based on the study of Younes et al. [18] and using PASS version 11 (NCSS, LLC. Kaysville, Utah, USA) with 80% power at the 5% level of significance (α = 0.05).

The subjects were selected by stratified random sampling among students from 10 kindergarten, 10 elementary school and 10 middle and high schools evenly distributed in four regions of the city. At least five students were randomly selected at each school. A total of 261 subjects were participated in study.

The study samples were male and female students aged 5–18 years, who had maxillary molar and canine bilaterally and normal occlusion. Normal occlusion was considered as dental and skeletal Class I occlusion with a satisfactory clinical occlusion [1, 38]. Individuals with apparent caries, facial asymmetry, oral habits, dental abrasions, palatal cleft, craniofacial syndrome, history of maxillofacial and dental fracture, and previous orthodontic therapy were excluded. Furthermore, subjects with primary canine or molars in one side of the arch and permanent teeth erupted on the other side were excluded.

Demographic information including age and gender were recorded, and the dentition stage was clinically determined for each subject by a trained examiner. Then, dental impressions were taken from all participants by alginate (Orthoprint, Zhermack Spa, Italy). Impression were poured by orthodontic stone (Orthodontic model mix stone, Kerr, Switzerland) and the dental casts were prepared.

The casts were classified according to dentition stage as: Primary stage (no permanent dentition erupted), Mixed stage (at least one or more primary teeth and at least one permanent tooth), and Permanent stage (permanent dentition with no primary teeth).

For each cast, palatal width and height were measured as follows: [1, 28, 39].

• Palatal inter-molar width:
-  º Primary dentition: the linear distance between the midpoint of central fissure in primary maxillary second molars for Korkhaus compass; and distal of primary maxillary second molars on the gingiva for digital caliper
-  º Mixed and Permanent dentition: the linear distance between the midpoint of central fissure in permanent maxillary first molars for Korkhaus compass and apex of interdental papillas between permanent second premolar and permanent first molar for digital caliper
• Palatal inter-canine width:
-  º Primary and Mixed dentition: the linear distance between deciduous canine cusp tips
-  º Permanent dentition: the linear distance between permanent canine cusp tips
• Palatal height at molar area:
-  º Primary dentition: the perpendicular distance from a line drawn from the distal margin of second primary molars to the palatal vault in the midline
-  º Mixed and permanent dentition: the perpendicular distance from a line drawn from the distal margin of permanent first molars to the palatal vault in the midline
• Palatal height at canine area:
-  º Primary dentition: the perpendicular distance from a line drawn from the deciduous canine cusp tip to the palatal vault in the midline
-  º Mixed and permanent dentition: the perpendicular distance from a line drawn from the permanent canine cusp tip to the palatal vault in the midline

The measurements were done by Korkhaus’ compass (Dentaurum, Germany) with accuracy of 0.5 mm for molar area; and by digital caliper (Mitutoyo Corp., Japan) with accuracy 0.01 mm for molar and canine areas. Owing to lack of height adjustability, Korkhaus’ compass could not be used for canine area.

The palatal height index is calculated from Korkhaus’ compass measurement as follows:

$$ \mathrm{palatal}\ \mathrm{height}\ \mathrm{index}=\frac{\mathrm{palatal}\ \mathrm{height}\ }{\mathrm{palatal}\ \mathrm{width}} \times 100 $$

Intra-observer reliability was determined using intra-class correlation coefficient test (ICC). Thirty casts (> 10% of total sample) were randomly selected and evaluated one week later by the same operator. Intra-observer reliability was considered excellent with ICC value of 0.89.

Data were analyzed using SPSS 15 (SPSS Inc., Chicago, IL, USA). One-way ANOVA followed by Tukey’s post hoc test and t- test were applied to verify the existence of significant differences in variables among dentition stages and between male and females, respectively. The level of significance was set at *p* <  0.05.

## Results

A total of 261 subjects participated in this study. Those casts with fracture or presence of bubble on the canine cusp tips or the central fissure in molars were excluded. Therefore, analyses were done for 237 with primary (21.5%: 24 m, 27 f), mixed (21.9%: 22 m, 30 f) and permanent (56.5%: 61 m, 73 f) dentitions. The mean age of the children at primary dentition, mixed dentition and permanent dentition stages for both genders presented at Table 1.

**Table 1:** Descriptive statistic for age among primary, mixed and permanent dentitions

	Male	female	total
primary	5.46 ± .51	5.59 ± 0.5	5.53 ± .50
Mixed	9.14 ± 1.32	8.73 ± 1.12	8.9 ± 1.26
Permanent	14.84 ± 1.97	14.95 ± 1.87	14.9 ± 1.91

Mean ± standard deviation are presented

Palatal width revealed a significant difference among primary, mixed and permanent dentitions. (*p* <  0.001) Palatal inter-molar width progressively increased from primary to mixed and permanent stage. Palatal inter-canine width increased from mixed to permanent stage. Palatal width was significantly higher in males than in females. (*p* <  0.05) (Table 2).

**Table 2:** Comparison of palatal width among primary, mixed and permanent dentitions; and between males and females

	Mean ± SD of palatal width (mm)								
	inter-molar/Korkhaus’ compass			inter-molar/digital caliper			inter-canine/digital caliper		
	total	male	female	total	male	female	total	male	female
primary	39.37^a^ ± 1.81	39.50^a^ ± 1.96	39.20^a^ ± 1.71	38.55^a^ ± 3.45	38.01^a^ ± 4.36	39.31^a^ ± 1.56	28.47^a^ ± 3.13	28.38^a^ ± 3.90	28.59^a^ ± 1.84
Mixed	44.90^b^ ± 5.36	43.95^b^ ± 7.86	45.60^b^ ± 2.15	44.75^b^ ± 6.78	43.85^b^ ± 10.12	45.42^b^ ± 2.37	28.94^a^ ± 8.73	29.31^a^ ± 7.13	28.68^a^ ± 9.85
Permanent	47.21^c^ ± 2.78	48.81^c^ ± 2.58	45.87^b^ ± 2.17	47.17^c^ ± 3.01	49.06^c^ ± 2.21	45.59^b^ ± 2.70	33.54^b^ ± 4.85	34.97^b^ ± 2.04	32.34^b^ ± 6.07
*P* value*	< 0.001	< 0.001	< 0.001	< 0.001	< 0.001	< 0.001	< 0.001	< 0.001	< 0.038
Total		46.58 ± 5.43	45.32 ± 2.73		46.54 ± 6.54	45.09 ± 3.01		32.87 ± 4.90	31.08 ± 7.28
*P* value**		0.031			0.036			0.025	

*using one-way ANOVA followed by Tukey’s post hoc test. Groups with the same letter are not significantly different

**t-test

Palatal height indicated a significant difference among primary, mixed and permanent dentitions at molar and at canine area (*p* <  0.001). There was no significant difference between males and females in palatal height at molar area measured by Korkhaus compass (*p* = 0.099) or digital caliper (*p* = 0.597). However, palatal height was significantly higher in males compared to females at canine area (*p* = 0.024) (Table 3).

**Table 3:** Comparison of palatal height among primary, mixed and permanent dentitions; and between males and females

	Mean ± SD of palatal height (mm)								
	molar area/Korkhaus’ compass			molar area/digital caliper			canine area/digital caliper		
	total	male	female	total	male	female	total	male	female
primary	11.16^a^ ± 1.63	10.90^a^ ± 1.35	11.51^a^ ± 1.99	15.08^a^ ± 1.31	15.07^a^ ± 1.61	15.10^a^ ± 0.85	7.77^c^ ± 3.54	9.63^c^ ± 1.79	5.21^b^ ± 0.65
Mixed	11.87^a^ ± 5.29	13.02^a^ ± 7.87	11.03^a^ ± 1.63	13.70^a^ ± 3.74	13.30^a^ ± 4.31	13.99^a^ ± 3.30	4.00^a^ ± 2.21	4.02^a^ ± 2.06	3.98^a^ ± 2.35
Permanent	15.55^b^ ± 2.46	15.91^b^ ± 2.4	15.24^b^ ± 2.48	19.38^b^ ± 2.45	19.74^b^ ± 2.45	19.09^b^ ± 2.42	6.23^b^ ± 1.84	6.88^b^ ± 2.03	5.69^b^ ± 1.48
*P* value*	< 0.001	< 0.001	< 0.001	< 0.001	< 0.001	< 0.001	< 0.001	< 0.043	< 0.001
Total		14.65 ± 4.61	13.83 ± 2.97		17.68 ± 4.06	17.42 ± 3.49		6.53 ± 6.38	5.19 ± 1.86
*P* value**		0.099			0.597			0.024	

*using one-way ANOVA followed by Tukey’s post hoc test. Groups with the same letter are not significantly different

**t-test

There was a significant difference in palatal height index among primary, mixed and permanent dentitions at molar (*p* <  0.001), and at canine area (*p* <  0.001). There was no significant difference between males and females in palatal height index at molar area (*p* > 0.5. Although, the index was significantly higher in males compared to females at canine area (*p* = 0.001) (Table 4).

**Table 4:** Comparison of palatal height index among primary, mixed and permanent dentitions; and between males and females

	Mean ± SD of palatal height (mm)								
	molar area/Korkhaus’ compass			molar area/digital caliper			canine area/digital caliper		
	total	male	female	total	male	female	total	male	female
primary	27.53^b^ ± 3.00	27.10^b^ ± 3.10	28.12^b^ ± 2.96	38.90^b^ ± 5.11	39.23^b^ ± 6.49	38.46^b^ ± 2.60	16.61^ab^ ± 4.09	15.43^a^ ± 4.82	18.23^b^ ± 2.18
Mixed	24.48^a^ ± 3.79	24.86^a^ ± 4.28	24.19^a^ ± 3.43	33.38^a^ ± 6.28	33.94^a^ ± 7.47	32.97^a^ ± 5.34	13.30^a^ ± 5.29	15.57^a^ ± 4.81	11.64^a^ ± 5.07
Permanent	33.00^c^ ± 5.51	32.53^c^ ± 5.26	33.42^c^ ± 5.71	41.50^b^ ± 6.44	40.90^b^ ± 6.76	42.00^c^ ± 6.17	18.20^b^ ± 4.74	19.32^b^ ± 4.64	17.27^b^ ± 4.65
*P* value*	< 0.001	< 0.001	< 0.001	< 0.001	< 0.001	< 0.001	< 0.001	< 0.001	< 0.001
Total		30.10 ± 5.87	30.54 ± 6.47		39.08 ± 7.42	39.30 ± 6.98		17.99 ± 4.99	15.81 ± 5.28
*P* value**		0.588			0.815			0.001	

*using one-way ANOVA followed by Tukey’s post hoc test. Groups with the same letter are not significantly different

**t-test

## Discussion

The development of the dental arches is a continuous process with some changes during the mixed developmental period [1]. Between adolescence and adulthood, we found a slow continuous change in all dimensions. These findings are of importance for orthodontic diagnosis and treatment planning, as well as for post-treatment stability [1, 10]. Most cephalometric and anthropometric norms in Iranian population are significantly different from the published norms [31–37]. This study aimed to evaluate the palatal dimensions at different stages of dentition in Iranian children and adolescent with normal occlusion.

The results of this study demonstrated significant changes in the dentition from the primary until the permanent stage. Our results showed that palate and alveolar process exhibit a growth pattern that is similar and concurrent with other part of face. The different palatal growth in two genders is the result of different growth spurt pattern [3, 5–7, 31].

In our study, generally, palatal width increased in both site of inter-molar and inter-canine during transition of primary to permanent dentition. Increase of the maxillary dental arch width during these periods explained by two mechanism. First, growth of alveolar process in all dimension. Second, alveolar processes diverge as the permanent teeth erupt [8].

The inter-molar palatal width in males significantly increased with advancing dentition from primary to permanent stages (*p* <  0.001).Similar growth pattern in other parts of Iranian face has been published [31]. However, in females there is a considerable increase in inter-molar width from primary to mixed stage followed by little increase into permanent stage. Females experience the juvenile growth spurt at an earlier stage of the life than males do. This growth period is significantly shorter in females than in males. Palatal growth pattern follows the same pattern in either gender. The difference between two sexes in our study should be interpreted in this context. Hassanali and Odhiambo found inter-molar palatal width increase in both genders with advancing age from 6 to 12 years [28]. A study on normal Brazilian children by Louly et al. revealed no significant change in inter-molar width between ages of 9 and 12 [40]. These studies are in contrast with ours. A longitudinal study showed a gradual increase in the inter-molar width that attained a stable condition at about 12 years with no further significant changes [11].

According to the literature, the inter-molar palatal width is higher in males than in females (*p* <  0.05) [11, 24, 41–43]. However, another study suggested no statistically significant sexual dimorphism in inter-molar width [40].

The present study showed a stable inter-canine palatal width from primary to mixed stages. A rapid increase at inter-canine widths occurred from primary to mixed stage in several studies [14–16]. Šlaj et al. reported a slight inter-canine width decrease from early to late mixed dentition [13]. These studies are in contrast with our results.

Inter-canine width was found to have a slight increase from mixed dentition toward the permanent stage in both genders in our study. Louly et al. concluded that the slight increase of the inter-canine width from 9 to 12 years old was not significant [40]. On the contrary, Moorrees and Tsujino and Machida reported a decrease from the mixed to the permanent stage [12, 16].

Racial differences may partly explain these controversies. Like many other anthropometric measurements that vary from one ethnical group to other, palatal width appears to be different in various part of the world.

Palatal height increased during transition of dentition due to vertical growth of alveolar process and eruption of teeth [8]. This study indicated that the palatal height at molar area increased from mixed to permanent stage. Thilander reported the height increase from 5 to 16-year-old [1].

There was no significant difference in palatal height at molar area in males compared to females (*p* > 0.05). Similar to our study, Tsai and Tan found no significant difference in palatal height between both sexes [24]. In contrast to our results, Al-Zubair [41] and Thilander [1] showed that palatal height in molar site was larger in females than males. This contradiction can be attributed to ethnic differences among studied groups.

The palatal height at canine area decreased from primary to mixed and increased from mixed to permanent stage. These changes, however, were greater in males than in females (*p* <  0.05). Louly et al. reported non-significant increase of palatal height from 9 to 12 years old with statistically significant sexual dimorphism at age of 10 [40]. Thilander described the continuous increase of palatal height at this period. He suggested “With premolars and molars in occlusion, there should not be any further increase of the alveolar process and hence no further increase of palatal height. On the other hand, continuous remodeling of the palate with bone deposition orally should decrease this distance. Furthermore, tooth wear is a common occurrence with increasing age, which also should decrease palatal height. However, the continuous increase of palatal height seems to be an effect of a slow continuous eruption of the teeth” [1]. This concept may explain the palatal height increase from mixed to permanent dentition in our study sample.

The palatal height index decreased from primary to mixed stages and then increased to permanent stage in males and females. These changes roughly correspond to pattern of palatal height changes. A similar pattern of was reported by Younes et al. [18]. Our findings are in contrast to previous studies that showed a progressive increase during the growth period [18, 26, 27, 44]. Furthermore, our study exhibited the values of palatal height index with a substantial inconsistency to other studies [18, 25, 45]. These controversial results can be attributed to differences in studies’ population and methodology.

There was no significant difference in palatal height index at molar area between males and females. However, the index at canine area was significantly higher in males compared to females, presenting the sexual dimorphism at this area.

## Conclusions

Our results showed that palatal width of males significantly higher than females. Palatal inter-molar width progressively increased from primary to mixed and permanent stage. Palatal inter-canine width increased from mixed to permanent stage. Palatal height and palatal height index decrease from primary to mixed dentation, then increase from mixed to permanent dentition. Palatal height and palatal height index at canine area were significantly higher in males compared to females, but these values did not show significant difference at molar area. Congruent to other investigation, our study revealed the influence of sex and ethnicity on palatal dimension.

These values could be considered as baseline for oral development studies and orthodontic treatment planning among Iranian children and adolescent. However, further longitudinal studies are required.
